# Supplementary material for: Structure and DNA-bridging activity of the essential Rec114–Mei4 trimer interface
Source: Genes Dev. 2023 Jun 1;37(11-12):518–34. doi: 10.1101/gad.350461.123 (PMC10393192; doi:10.1101/gad.350461.123)
Supplement: Supplemental Material [file supp_37_11-12_518__DC1.html]

Structure and DNA-bridging activity of the essential Rec114–Mei4 trimer interface — Structure and DNA-bridging activity of the essential Rec114–Mei4 trimer interface — Supplemental Material 

# Structure and DNA-bridging activity of the essential Rec114–Mei4 trimer interface

## Supplemental Material

- Supp\_movie\_1.avi
- supp\_movie\_2.avi
- Supp\_movie\_3.avi
- Supp\_movie\_4.avi
- Supp\_movie\_5.avi
- Supplemental\_combined.pdf
